# Enhanced Motor Function by Training in Spinal Cord Contused Rats Following Radiation Therapy

**DOI:** 10.1371/journal.pone.0006862

**Published:** 2009-08-31

**Authors:** Ronaldo Ichiyama, Melissa Potuzak, Marissa Balak, Nurit Kalderon, V. Reggie Edgerton

**Affiliations:** 1 Departments of Physiological Science and Neurobiology, University of California Los Angeles, Los Angeles, California, United States of America; 2 Brain Research Institute, University of California Los Angeles, Los Angeles, California, United States of America; 3 Sloan-Kettering Institute for Cancer Research, Molecular Pharmacology & Chemistry Program, New York, New York, United States of America; Universidade Federal do Rio de Janeiro (UFRJ), Instituto de Biofísica da UFRJ, Brazil

## Abstract

Weight-bearing stepping, without supraspinal re-connectivity, can be attained by treadmill training in an animal whose spinal cord has been completely transected at the lower thoracic level. Repair of damaged tissue and of supraspinal connectivity/circuitry following spinal cord injury in rat can be achieved by specific cell elimination with radiation therapy of the lesion site delivered within a critical time window, 2-3 weeks postinjury. Here we examined the effects of training in the repaired spinal cord following clinical radiation therapy. Studies were performed in a severe rat spinal cord contusion injury model, one similar to fracture/crush injuries in humans; the injury was at the lower thoracic level and the training was a combined hindlimb standing and stepping protocol. Radiotherapy, in a similar manner to that reported previously, resulted in a significant level of tissue repair/preservation at the lesion site. Training in the irradiated group, as determined by limb kinematics tests, resulted in functional improvements that were significant for standing and stepping capacity, and yielded a significant direct correlation between standing and stepping performance. In contrast, the training in the unirradiated group resulted in no apparent beneficial effects, and yielded an inverse correlation between standing and stepping performance, e.g., subject with good standing showed poor stepping capacity. Further, without any training, a differential functional change was observed in the irradiated group; standing capacity was significantly inhibited while stepping showed a slight trend of improvement compared with the unirradiated group. These data suggest that following repair by radiation therapy the spinal circuitries which control posture and locomotor were modified, and that the beneficial functional modulation of these circuitries is use dependent. Further, for restoring beneficial motor function following radiotherapy, training seems to be crucial.

## Introduction

Spinal cord injury results in damage to the vasculature and blood supply and in destruction of the brain-spinal cord fiber connectivity, leading to progressive tissue decay at the damage site and paralysis below the lesion site. Lack of wound repair and chronic tissue decay accompanied by enlarging cavitation at the lesion site are characteristic of the pathology of spinal cord injury [1]–[5]. The severed supraspinal fibers fail to cross the widening wound gap and brain motor control distal to the lesion site is impaired.

However, it is possible, without having supraspinal reconnectivity, to attain some locomotor control in animal models [6] and in human [7]–[8] by load bearing step training. This motor function is mediated via local spinal cord circuitry. The lumbar spinal cord can learn to stand [9]–[10] and to step [6], [9]–[18] in the absence of supraspinal input. Daily treadmill training of adult cats whose spinal cord is completely transected at the low-thoracic level can attain a significant level of control of coordinated and full weight-bearing steps [6], [14], [16].

Recent studies [3] suggest that normal wound healing processes take place during the first 2 weeks after injury and that the onset of tissue decay occurs towards the end of the second week and/or during the third week postinjury (e.g., see Fig. 2D in previous study [3]). The pathologic outcome of spinal cord transection and contusion injury can be manipulated and intrinsic wound repair obtained by specific elimination of some cells generated at the lesion site [3], [19]–[20]. Specifically, localized radiation therapy ―as used for curing cancer― given at the lesion site within a critical time window, 2–3 weeks postinjury, seems for the most part to halt the onset and progression of decay processes; consequently some repair is facilitated in transected [3], [5], [21] and in the severely contused [20] rat spinal cord.

Associated with wound repair, some of the transected supraspinal fibers can re-grow across the lesion site into the distal stump as shown qualitatively and quantitatively by antero- and retrograde axonal tracing [3], recovered some of the disrupted circuitry as demonstrated by electrophysiology [21] and regained some control of hindlimb (HL) muscle function [5], [21].

Here, we examined the outcome of standing and stepping training in a low-thoracic spinal cord injured rats in which the intrinsic wound repair was made possible by radiation therapy. To differentiate effects due to the radiotherapy we examined the functional outcomes of the training protocol in spinal cord injured rats that were previously irradiated or unirradiated. The study was conducted in a model similar to fracture/crush spinal cord injuries in humans [22]–[23] in severe contusion injury; this injury leaves some of the supraspinal and ascending fiber tracts intact [24]–[25]. Since the effects of training have been studied primarily in complete transection models, this study provides also novel information about the effects of motor training in severe contusion injury.

## Materials and Methods

### Surgical procedures

Adult Sprague-Dawley female rats (Charles River Breeding Laboratories), 3–5 mos old (average weight, 300±22 g) were used. Laminectomy was performed at T10 under anesthesia as described previously [3], [20]. Severe contusion injury was performed with a weight-drop device (2.5 mm in diameter) [22], dropping it from 50 mm on the exposed spinal cord surface [24]. A midline incision, to prevent fluid buildup and secondary damage due to hemorrhage was performed 2 h postinjury by a perpendicular stabbing at 5 points along the midline of the lesion site with a 26G needle as previously described [20]. No special care was needed apart from bladder expression, which were emptied manually 3–4 times a day within the 1st week postinjury and 1–2 times per day thereafter. A total of 56 severely contused rats were prepared at the Kalderon laboratory at Sloan-Kettering Institute (SKI) and shipped (early 4th week postinjury to the Edgerton laboratory at University of California at Los Angeles (UCLA) for the training portion of the study. The animal care was in accordance with the Institutional Animal Care and Use Committee guidelines at SKI and as approved by the Chancellor's Animal Research Committee at UCLA.

### Radiation therapy

Irradiation was performed at the Kalderon laboratory; it was delivered by an x-ray generator (XRad 320, Precision X-ray, East Haven, CT), an orthovoltage unit operating at 250 kVp, 12 mA with 0.25 mm Cu filtration, at a dose rate 101.7 cGy/min, at a distance of 50 cm from the skin. Treatment was delivered through a posterior approach, centered at the site of lesion and exposure field of 25 mm×20 mm (long×wide) as previously described [5]. Radiotherapy was about half the clinical level [5], [19]; a total dose of 20 Gy was given in 10 fractions of 2 Gy per day which were delivered daily for 5 consecutive days, resting 2 days and then resuming for 5 more consecutive days, starting on day 12 postinjury. Selection for irradiation was random and a total of 28 contused rats were irradiated.

### Screening of motor function

General locomotor behavior to assess the outcome of the injury was visually monitored according to the Basso, Beattie and Bresnahan (BBB) open field test [26]. Locomotor behavior was tested in the first 2 weeks postinjury once every 2–4 days and later once a week until shipped to UCLA. At the Edgerton laboratory the BBB scores were determined on the day of assignment for training for pairing purposes as explained below and then once every 2–3 weeks.

### Animal care at UCLA

Until the end of the study the Edgerton Laboratory was blinded as to the identity of the irradiated rats, i.e. the rats were shipped as groups A and B. Upon arrival, each of the rats was housed in individual large cage, and bladder expressions were performed 2–3 times per day. Because some of the rats exhibited spastically flexed HLs which made it difficult to train them, all 56 rats received HL physical therapy for 2 min per day, starting a day after arrival at UCLA and continuing until the end of the study (90–93 days postinjury). The physical therapy of each HL by hand consisted of: gentle muscle massages and a passive range of HL joints' motion similar to locomotion.

### Locomotor training

Training of the rats was started at about 1 week after arrival at UCLA (30 days postinjury). The irradiated and unirradiated rats were randomly assigned to training resulting in 4 groups (14 rats/group): 1) unirradiated; 2) trained unirradiated; 3) irradiated; and 4) trained irradiated. Because of the variability/asymmetry in the behavioral outcome of severe contusion, selection for training was done by pairing irradiated with unirradiated rats that have similar BBB scores (similar motor deficits) as determined on that day. In retrospect, the profiles of the BBB scores of the 4 groups at shipping time from SKI to UCLA (21–24 days postinjury) were similar, showing lack of any bias in assignment for treatments. These paired rats were trained simultaneously in a dual treadmill set-up (as explained below). Training included both standing and bipedal stepping, given daily (5 days per week) for 20-min per session for 8 weeks. For training the HLs, the rats were suspended/supported over the treadmill belt with an upperbody harness connected to a body weight-support apparatus consisting of a lever with counter weights [27]. Each training session was started by placing the rats in a static standing position for 1 min, while bearing the maximal possible weight on their plantar paws' surface before collapsing [10]. This was followed by step training. The rats were progressively trained to step for as long as they could at the fastest speed; i.e., starting the treadmill at 6 cm/s and gradually increasing to 13.5 and 21 cm/s speed. When one of the rats in the pair failed to step, the treadmill was stopped and the pair stood for 1 min, as described above. Altogether, the protocol design was such that its progression, and the time spent stepping or standing, was dependent upon the performance of the weaker rat of the pair. No noxious stimuli (e.g., tail pinching) were utilized during the training.

### Stand and step testing

Testing for standing and stepping was performed with an automated, computer controlled, body weight support apparatus associated with a treadmill using an upper body harness [28]. Stepping and standing during the test was captured by 6 video cameras at a rate of 60 frames/s; images were processed with Peak Motus 8.0 (Peak Performance Technologies, Inc., Centennial, CO) to create 3D reconstructions of stepping kinematics. Testing was performed on all 56 rats, (trained and non-trained) on the corresponding last day of the 8-week training protocol. Before testing, each rat was bipedally stepped for 5 min. After shaving of the rats' HLs 5 retro-reflective markers (4 mm diameter) were placed over the: iliac crest; trochanter of the femur; lateral patella; lateral maleolus; and metatarsal-proximal phalange joint. Testing for stepping included 1 min continuous stepping sessions at 6, 13.5 and 21 cm/s speeds (from slow to fast), with 1 min of rest/stand in between speeds. The total number of steps performed was computed from the 30 s of best continuous stepping period within the 1-min session. Testing for standing directly followed the stepping test. For the standing test, the plantar surface of the hind paws was placed in contact with the surface of the stationary treadmill belt. The body weight bearing ability was measured by decreasing the support every 10s in 10% decrements, starting at 100% support and ending at 0%. After every decrease in weight support the rat was lifted and repositioned in a standing posture. The percentage of the body weight support at which the rat collapsed was recorded. Collapse was determined visually when the rat's HLs were completely flexed and the hind quarters touched the treadmill belt. This criterion was verified by joint angular kinematics.

### Tissue harvest and histology

Harvest of cord tissue samples containing the lesion site, i.e., tissue fixation and removal from the vertebrae were performed, as previously described [3], at UCLA and were frozen there. Frozen cord samples were cryostat-sectioned (15 µm thick) in a sagittal plane at Kalderon's laboratory; the serially collected sections were thionin stained and examined by light microscopy.

### Quantitative morphometry

Quantitative analysis for degree of tissue repair/preservation was performed by morphometry of the acquired light microscope digital images of the lesion site with Image-Pro® Plus 5 software (Media Cybernetics, Silver Spring, MD) as previously described [20]. The entire process was blinded. Briefly, the pertinent cord sections were digitally photographed when the lesion site was at the center of the field of view and the area of normal and non-decaying tissue was measured. Quantitative analysis was done in a total of 6 sequential sections that were about 0.16 mm apart from each other and which were collected from the central portion of the cord (sagittal) representing about 50–60% of the lesion site. The percent of tissue spared was calculated in respect to equivalent volume measured in normal intact cord (n = 3). The morphometry was performed only on 24 rats (6 per group) because of technical problems in tissue preservation.

### Statistical Analysis

Morphometric data were analyzed using the t-test and locomotor behavior data using the non-parametric, Mann-Whitney test. Some additional analysis of the stepping and knee angle data was performed by first ranking the relevant data-values of the 4 groups together (of 56 rats) according to the Kruskal-Wallis ranking. These statistical analyses including the pairs' comparison of correlation coefficients were performed using Systat for Windows, version 11 (Systat Inc). Additional overall statistical analyses were performed by UCLA and SKI statisticians using the relevant procedures (SAS software) as follows: overall means of knee angle, overall standing capacity and overall stepping incidence for the 4 groups (at UCLA) and the overall difference in the correlation coefficients among the 4 groups (at SKI). Significance levels were considered at *p*<0.05.

## Results

### Wound repair by radiation therapy

**Figure 1:**
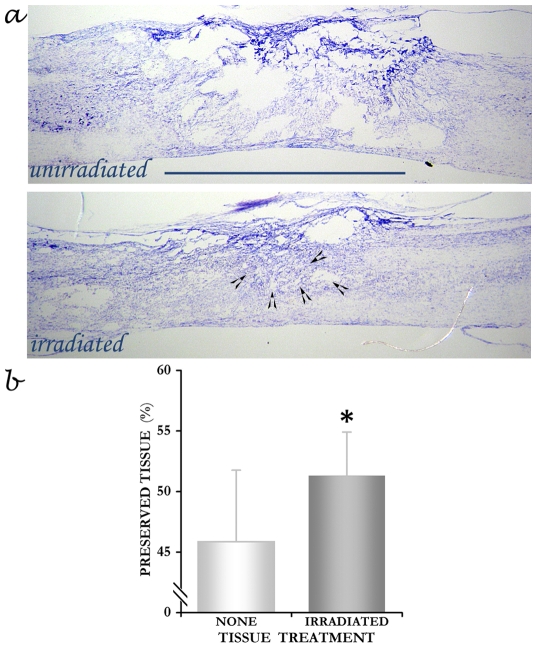
Structural repair by radiation therapy. *a*. Micrographs of thionin-stained sagittal sections through the epicenter of 2 differently treated severely contused cords: unirradiated and irradiated, seen 90 days PI. Note, the cavitation in the untreated vs. the substantial wound repair and the small abnormal area (arrowheads) in the irradiated cord. Bar = 3 mm. *b*. Degree of tissue preservation/repair, as measured by area of remaining tissue, in the unirradiated and irradiated cords (expressed as % of intact spinal cord tissue). Note, a significant (*p* = 0.015) degree of tissue preservation in the irradiated group. However, the actual degree of tissue preservation/repair by the radiotherapy is much higher because our measurements for tissue decay included about 40–50% of intact normal tissue. Therefore, the calculations of degree of repair were skewed downward, which tends to deemphasize the actual repair. Significance * *p*<0.05; error bars, SD.

Radiation therapy resulted in wound healing and repair (Fig. 1a) very similar to that reported previously [3], [20]. Quantitative morphometry of the lesion site showed significant (*p* = 0.015) degree of tissue preservation and/or tissue repair in the irradiated as compared with the unirradiated rats (Fig. 1b). It should be noted that the actual degree of tissue preservation/repair by the radiotherapy is by far much higher than the quantitative statistical significance shown in figure 1b.

### Training the repaired spinal cord to stand

**Figure 2:**
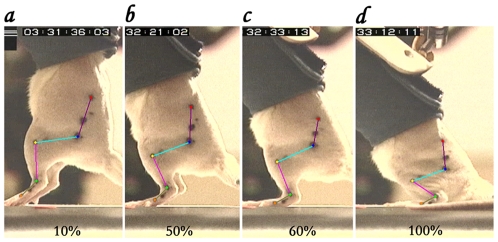
Knee angle as a measure for standing capacity. A stick diagram of the HL joints, at the hip, knee and ankle superimposed on the actual HL of one rat while under different levels of weight load: *a*. 10%, *b*. 50%, *c*. 60%, and *d*. 100% of body weight. Note, the gradual decrease in knee angle with increased loading; this rat had a 90% weight bearing capacity, collapsing at 100% weight load.

HL standing performance improved significantly in the irradiated rats that were trained. The capacity of the HL to stand (bear its body-weight) was determined by measuring the changes in knee angle when increasing load-bearing in increments of 10% starting from 0% level (Fig. 2). With increasing load the knee angle gets smaller (Fig. 2a–c); it was shown that at a given load the knee angle is greater in those animals that can bear more weight [28].

**Figure 3:**
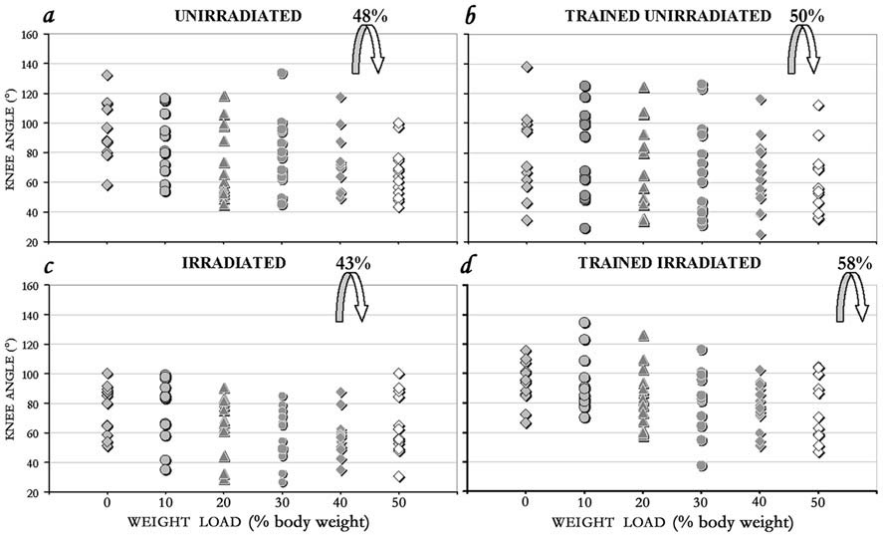
Individual standing capacity of all rats. Scatterplot of the knee angles under increasing weight bearing conditions of each of the rats (individual symbol) in the respective 4 treatment groups: *a*. unirradiated; *b*. trained unirradiated; *c*. irradiated; and *d*. irradiated trained. The tested loads are expressed as the percentage of body weight shown in 10% increments from 0 to 50%. The arrows point to the average collapse level; the best weight support capacity was in the irradiated trained group at 58%.

The changes in knee angle with the increasing load for each rat and the mean collapse level of the treatment groups are summarized (Fig. 3). By overall analysis, the knee angle was significantly correlated (*p*<0.0001) to the load placed on the HL. Each treatment group had a different capacity to bear its weight (collapse level): 58% in the irradiated trained (Fig. 3d), 50% in the unirradiated trained (Fig. 3b), 48% in the unirradiated (Fig. 3a) and 43% in the irradiated group (Fig. 3c).

The overall means of the knee angle, measured at all levels of weight support conditions, (±SE) of the 4 groups were: 78.7±5.4 (unirradiated); 71.6±5.2 (trained unirradiated); 66.1±5.4 (irradiated); and 83.6±5.2 (trained irradiated). Accordingly, standing capacity of the trained irradiated was significantly (*p* = 0.02) better than that of the irradiated group, while differences between the other groups were not significant.

**Figure 4:**
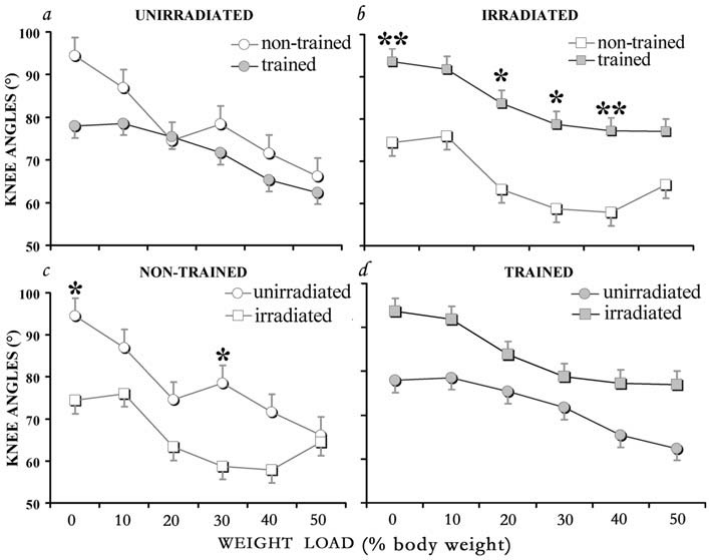
Comparative standing capacity under different treatments. The average knee angle at each load (expressed as % of body weight) put on the rats under 4 treatments: *a*. unirradiated; *b*. irradiated; *c*. non-trained; and *d*. trained. Comparison between the treatment groups was performed with the Kruskal-Wallis one-way ranking test; significant differences in standing capacity were observed: due to training in the irradiated rats (*b*); and without training between irradiated and unirradiated groups (*c*). Significance * *p*<0.05; ** *p*<0.01. Error bars, SE.

Comparison of the standing performance as defined by the average of knee angles at each load of the 4 groups (Fig. 4), performed by the non-parametric Kruskal-Wallis one-way ranking test, shows that in the trained irradiated rats the knee angle was significantly larger at 0%, 20%, 30% and 40% load levels than that of the irradiated group without training (Fig. 4*b*). Some significant differences in standing were associated not with training but with the radiotherapy (Fig. 4*c*). Without training, the knee angle in the irradiated was significantly lower (reduced standing) from that of the unirradiated group at 0% and 30% body-weight bearing (Fig. 4*c*), suggesting some changes in circuitry that are due to the radiotherapy. Altogether, in all tests we used, the best standing performance was always seen in the irradiated trained rats.

### Training the repaired spinal cord to step

**Figure 5:**
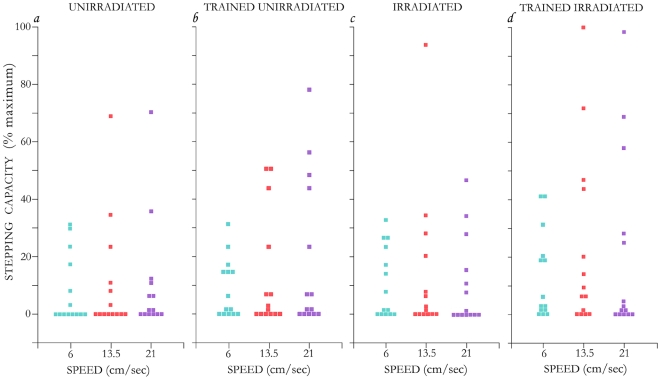
Individual stepping performance in all rats. Scatterplot of number of steps at 3 different speeds of each individual rat (single symbol) in the 4 treatment groups. The number of steps was normalized to the largest number of steps, 32, taken at any of the tests. Note, the difference in incidence of steppers between the groups, the best being that of the trained irradiated.

Stepping performance was assessed by counting the total number of plantar placed steps made within a 30 s testing period at 6, 13.5 and 21 cm/s (Fig. 5); analyzing for two stepping parameters, for incidence of capacity of stepping (% of rats taking at least one step), and for degree/level of stepping (number of steps made) within the treatment group. Altogether, incidence and degree of stepping were the highest in the irradiated trained at 6 and 13.5 cm/s; however, these differences were only significant for stepping incidence. For illustration of the effect of training on stepping capacity, see Supplemental Information which includes four video clips of the testing for stepping in irradiated rat (Video S1 and Video S3) *vs*. irradiated and trained rat (Video S2 & Video S4) at 6 and 13.5 cm/s speeds, respectively,

In each group some of the rats were unable to make even one step, i.e., they lacked stepping capacity, (Fig. 5). The best group across the 3 speeds with the highest incidence of stepping capacity was the irradiated trained with a range, across the 3 speeds, of 78%–64% success vs. 64%–50% in the irradiated non-trained, 64%–57% in the unirradiated trained, and 43%–57% in the unirradiated non-trained. Stepping incidence in the irradiated trained at 6 cm/s was (78%) substantially higher (*p* = 0.05) than that of the unirradiated non-trained rats (43%), while the differences in the other groups and/or at other speeds were not significant.

As for stepping degree, the irradiated trained group, at 6 and 13.5 cm/s speeds, had the best, while the unirradiated without training (at all 3 speeds) had the lowest level. The mean of step-ranking (based on the number of steps) at 6 cm/s was: 33 (irradiated trained); 29.5 (irradiated); 27.5 (unirradiated trained); and 24 (unirradiated); however, these differences were not significant [illustration of the effect of training, see Supplemental Information Videos S1-[Supplementary-material pone.0006862.s002] [Supplementary-material pone.0006862.s003] S4].

**Figure 6:**
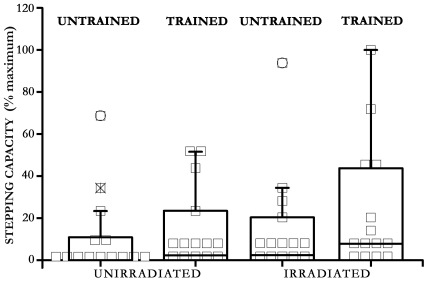
Comparative group stepping performance. Box-plot analysis (with Systat software) of stepping level (% of maximal number of steps) at 13.5 cm/min in: unirradiated; trained unirradiated; irradiated; and trained irradiated. Note, the floating points are the outlier values in each group; without these outliers, stepping level in the trained irradiated is significantly (*p* = 0.04) higher than that of the unirradiated group.

Significant differences between the 4 treatments can be seen in the level of stepping by the box plot analysis which identifies the outliers within each of the groups (Fig. 6). If the outliers are excluded, the level of stepping in the trained irradiated at 13.5 cm/s is significantly better (*p* = 0.04) than that of the non-trained group.

Finally, without training, standing and stepping capacities were contrasting in the irradiated group; in this group standing was lower (Fig. 4*c*) while the stepping function tended to be higher than that of the unirradiated nontrained group (Figs. 5–6).

### Enhanced motor function by training in the irradiated rats

**Figure 7:**
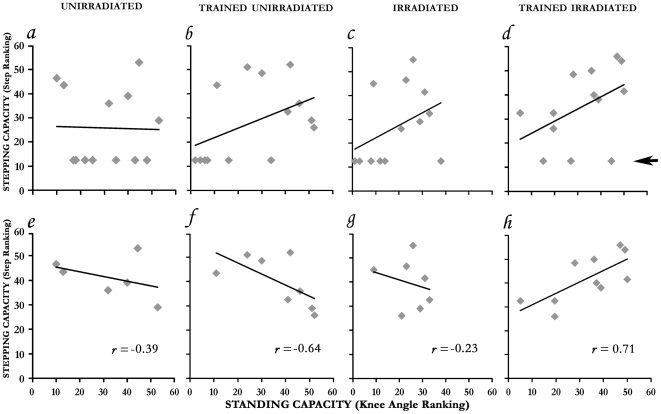
Correlation between stepping and standing performance. Correlation coefficients between the stepping and standing (knee angle) level at 13.5 cm/sec and 30% of body-weight bearing, respectively (conditions in which optimal functional outcomes were obtained). The ranking values are by Kruskal-Wallis ranking within the 4 groups: unirradiated (*a,e*); trained unirradiated (*b,f*); irradiated (*c,g*); and trained irradiated (*d,h*). The top panels (*a–d*) show data of all animals, arrow on the right margin points to the subgroup, aligned parallel to the x-axis, that failed to step (12.5 ranking = 0 steps); the bottom panels (*e–h*) show data without the subgroup that failed to step. Note, the trained irradiated (*h*) had a significant positive correlation (*r* = 0.71, *p* = 0.02) between stepping and standing ability whereas the trained unirradiated group (*f*) had an inverse, negative correlation (*r* = −0.64, *p* = 0.08).

The overall outcome of training was assessed by analyzing the relationship (correlation coefficient) between standing and stepping performance in each of the 4 treatment groups (Fig. 7). This analysis showed that the training elicited completely different effects in the irradiated from those in the unirradiated rats. While a significant direct correlation between the standing and stepping degree was found in the irradiated trained, an inverse correlation was found in the unirradiated trained group.

The distinction between the groups becomes evident when analysis is focused on the degree of motor function (Fig. 7*e–h*), i.e., by omitting the rats that failed to make any steps (those aligned in parallel to the x-axis in Fig. 7*a–d*). First, the irradiated trained group had a significant positive correlation (*r* = 0.71, *p* = 0.02) between stepping and standing ability (Fig. 7*h*), whereas the unirradiated trained group had a negative correlation (*r* = −0.64, *p* = 0.08) (Fig. 7*f*). Next, by Pair Comparison analysis (Systat), the irradiated trained significantly (*p* = 0.005) differed from the unirradiated trained group; the trained irradiated also differed from the non-trained irradiated (*p* = 0.07) or the non-trained unirradiated rats (*p* = 0.06). Finally, overall analysis of the data of the 4 groups shows that the relationship between stepping and standing overall significantly differed (*p* = 0.02) among the 4 groups. Altogether, training in the irradiated resulted in enhancement of both stepping and standing, while in the unirradiated group its effects remain unclear.

## Discussion

The major conclusion is that a combined standing/stepping training protocol in severe spinal cord contusion injury had a significantly beneficial functional effect in the irradiated rats. The data imply that following repair by radiotherapy the spinal circuitries which control posture and locomotor were modified, and that the beneficial modulation is use dependent. This conclusion is based on several observations. First, radiation therapy as previously reported facilitated wound healing and tissue repair. Second, radiation therapy *per se*, without any training, led to some functional changes, i.e. standing ability was significantly reduced while stepping showed a slight trend of improvement compared with the unirradiated group. Third, in the unirradiated group the combined stand and step training protocol failed to elicit a net beneficial functional outcome, showing opposing effects, i.e., enhancing either stepping or standing capacity. Fourth, the training protocol improved standing and stepping in the irradiated group and these motor performances were significantly and positively correlated.

### Effects of training in severe contusion

This study provides novel information about the effects of training in severe contusion injury. In the unirradiated group, there was not conclusive evidence of any effect of the combined stand-and-step training protocol either in standing or stepping performance. However, the inverse correlation seen between standing and stepping capacity in individual rats suggests that there was a training effect, but it seemed to have elicited improvement in either stepping or standing ability. An inverse relationship was previously observed, albeit not with a combined step and stand training; completely spinalized cats that were taught only to stand acquired an excellent standing ability, but their stepping ability was very poor and vice versa [10].

A few HL training studies have been performed in a moderate contusion injury which results in moderate functional deficits e.g., [29]–[30]. However, those do not pertain to this study because of the fundamental difference in the functional deficits and the training protocol. Following a moderate contusion the HLs can stand and also perform partial weight-bearing steps [30]; whereas following severe contusion the HLs are practically paralysed and have no ability to generate any weight-supporting steps [26]. Our correlation data of the unirradiated trained rats suggest that with a dedicated training of weight-support only, the severely contused rats can regain some HL standing capacity.

### Repair and constructive modulation of neural network by radiation therapy

Morphometric analysis demonstrates that radiotherapy facilitated wound healing and repair in the severely contused cords in a similar manner observed previously in transection injury [3], [19] and in severe contusion [20]. Our data of weight bearing and stepping capacity lend support to the assumption that consequent to wound healing similar regenerative events as occur in transection [3], [19] would follow in contusion injury, namely, some axonal fiber regrowth and reconnectivity of damage circuitry might take place.

The data derived from the groups which were not trained highlight the effects of radiation therapy (wound repair) on HL motor performance. These data suggest that some formation/modulation of neuronal networks took place in the irradiated group, presumably following wound repair. While stepping performance appeared to be unaffected, standing capacity (knee angle) was reduced/inhibited following the radiation therapy if the injured cord.

### The beneficial outcome of training in the irradiated group

The apparent lack of training effects in the unirradiated contused rats contrasts with the significant effect of training seen in the irradiated rats. The most compelling evidence of significant beneficial effect of training in the irradiated group comes from the correlation analysis. The switch from an inverse correlation in the unirradiated to a direct correlation in the irradiated group suggests a fundamental difference in response to training between the two groups. This indicates that the neural control systems were substantially modified by the radiotherapy. Furthermore, the significant reduction in standing capacity in the irradiated untrained group suggests that repair or formation of new networks is not sufficient to exhibit enhanced function. Specifically, even if repair to the circuitry were optimal, for restoring a substantial level of motor function training may be crucial.

As for the mode of enhancement in the irradiated group, several mechanisms are possible for such an outcome. In contusion injury, unlike a complete transection, some of the supraspinal and ascending fiber tracts remain intact [24]–[25]. Also, changes to spinal circuitries can be made by lack of limb activity [31]–[32] or by the addition of activity through training [33]. At present it is unknown to what extent the observed functional changes are due to spinal or supraspinal components. The radiation therapy could have spared local/supraspinal fibers and/or facilitated regrowth of damaged local/supraspinal axons and/or repair of damaged circuitries. Altogether, spared/repaired fibers potentially may form several combinations of spinal or supraspinal neural control components. Any of these potential combinations could explain the beneficial effects of training in the irradiated rats. The present study provides no insight as to the potential mechanisms involved. However, it provides a proof of principle that training can enhance motor function following radiotherapy.

## Supporting Information

Video S1 Enhanced stepping by training in irradiated severely contused rat. Four video clips of the testing for stepping in irradiated rat (S1 & S3) vs. irradiated and trained rat (S2 & S4) at 6 and 13.5 cm/s treadmill speeds, respectively, are presented for comparison. Here shown the irradiated rat tested at 6 cm/s. While both rats can ‘walk’ at the slower speed (S1 & S2) the effect of training becomes more pronounced at the higher speed where the irradiated stumbles (S3) while the irradiated and trained rat (S4) continues to ‘walk’ comfortably for the entire testing period. Note, the difference in the paw placement, plantar vs. dorsal, between the trained and non-trained rat.
(6.98 MB AVI)

Video S2 Enhanced stepping by training in irradiated severely contused rat. Four video clips of the testing for stepping in irradiated rat (S1 & S3) vs. irradiated and trained rat (S2 & S4) at 6 and 13.5 cm/s treadmill speeds, respectively, are presented for comparison. Here shown the irradiated and trained rat tested at 6 cm/s. While both rats can ‘walk’ at the slower speed (S1 & S2) the effect of training becomes more pronounced at the higher speed where the irradiated stumbles (S3) while the irradiated and trained rat (S4) continues to ‘walk’ comfortably for the entire testing period. Note, the difference in the paw placement, plantar vs. dorsal, between the trained and non-trained rat.
(7.97 MB AVI)

Video S3 Enhanced stepping by training in irradiated severely contused rat. Four video clips of the testing for stepping in irradiated rat (S1 & S3) vs. irradiated and trained rat (S2 & S4) at 6 and 13.5 cm/s treadmill speeds, respectively, are presented for comparison. Here shown the irradiated rat tested at 13.5 cm/s. While both rats can ‘walk’ at the slower speed (S1 & S2) the effect of training becomes more pronounced at the higher speed where the irradiated stumbles (S3) while the irradiated and trained rat (S4) continues to ‘walk’ comfortably for the entire testing period. Note, the difference in the paw placement, plantar vs. dorsal, between the trained and non-trained rat.
(8.42 MB AVI)

Video S4 Enhanced stepping by training in irradiated severely contused rat. Four video clips of the testing for stepping in irradiated rat (S1 & S3) vs. irradiated and trained rat (S2 & S4) at 6 and 13.5 cm/s treadmill speeds, respectively, are presented for comparison. Here shown the irradiated and trained rat tested at 13.5 cm/s. While both rats can ‘walk’ at the slower speed (S1 & S2) the effect of training becomes more pronounced at the higher speed where the irradiated stumbles (S3) while the irradiated and trained rat (S4) continues to ‘walk’ comfortably for the entire testing period. Note, the difference in the paw placement, plantar vs. dorsal, between the trained and non-trained rat.
(52.88 MB AVI)
